# Dietary protein intake affects the association between urinary iodine and clinically relevant depression: Evidence from NHANES 2007–2018

**DOI:** 10.1002/fsn3.3429

**Published:** 2023-05-19

**Authors:** Xue Kong, Xia Shen, Long Yang, Yuan‐Yuan Liu, Xue Gu, Yan Kong

**Affiliations:** ^1^ Department of Laboratory Medicine The Affiliated Wuxi People's Hospital of Nanjing Medical University, Wuxi People's Hospital, Wuxi Medical Center, Nanjing Medical University Wuxi China; ^2^ Department of Nursing, Wuxi Medical College Jiangnan University Wuxi China; ^3^ College of Pediatrics Xinjiang Medical University Urumqi China; ^4^ Department of Radiation Oncology Affiliated Hospital of Jiangnan University Wuxi China

**Keywords:** adults, clinically relevant depression, CRD, protein intake, urinary iodine concentration, urinary iodine concentration

## Abstract

Both iodine concentration and protein intake are important nutritional factors that may influence the development of depressive symptoms. However, there are no studies on the effect of protein intake on the relationship between iodine concentration and the risk of depression. The study aimed to explore the relationship between iodine and the risk of clinically relevant depression (CRD) according to protein intake. This study analyzed the adults (≥18 years) who participated in the 2007–2018 National Health and Nutrition Cross‐sectional Survey (*N* = 10,462). CRD was assessed using the Patient Health Questionnaire (PHQ‐9). Protein intake was assessed using two 24‐h dietary recalls and urinary iodine concentration (UIC) was measured using inductively coupled plasma dynamic response cell mass spectrometry. Weighted multivariate logistic regression and restrictive cubic splines were performed to assess the relationship between UIC and CRD according to protein category (low protein intake <0.8 g/kg/day; high protein intake: ≥0.8 g/kg/day). After controlling for sociodemographic, behavioral, chronic diseases, and dietary factors, a positive correlation was observed between UIC (log10) and CRD (OR: 1.36, 95% CI: 1.026, 1.795). Low UIC (<100 μg/L) was associated with a lower prevalence of CRD (OR: 0.73, 95% CI: 0.533, 0.995) in high protein intake individuals, whereas this relationship did not exist in those with low protein intake. Moreover, restrictive cubic splines confirmed a near L‐shaped relationship between UIC and CRD in the low‐protein group (nonlinear *p* = .042) and a linear relationship between them in the high‐protein group (nonlinear *p* = .392). This study illustrates that protein intake affects the relationship between UIC and CRD. Combining lower UIC and high protein intake may help reduce the prevalence of CRD, which would have significant implications for managing patients with depressive CRD in the clinical setting.

## Graphical abstract

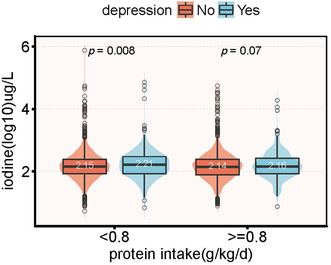

## 1 INTRODUCTION

Depression is a common disease globally and is estimated to affect 3.8% of the population, as well as 5% of adults (World Health Organization, 2022). A study on the incidence of depression in the US reported a high prevalence of depression (Cao et al., 2020). Research has shown that depression is a relapsing and chronic disease associated with the risk of mortality and metabolic disease, cardiovascular disease, and cancer mortality (Hidese et al., 2018; Inoue et al., 2018; Teng et al., 2006; Tran et al., 2017). In addition, depression is associated with a high risk of suicide, which is estimated to cause nearly 0.8 million deaths per year (Hawton et al., 2013). Therefore, identifying nutrients associated with depression is critical to preventing depression.

It has been suggested that neurobiological, social, and genetic factors are related to depression. Dietary intake is also a potentially modifiable factor of depression; for example, caffeine intake has been linked to the risk of depression (Iranpour & Sabour, 2019). Iodine is a trace element and an important component of the thyroid hormone (Zimmermann & Boelaert, 2015). At the same time, iodine can also increase the incidence of several chronic diseases (Liu et al., 2019; Scinicariello & Buser, 2018; Wang, Wan, Liu, Meng, Ren, et al., 2021; Wang, Wan, Liu, Meng, Zhang, et al., 2021). Recently, several studies have explored the relationship between iodine status and depression symptoms, with inconsistent findings. A study of 102 participants with euthyroid nodular goiter found that low urinary iodine concentrations may contribute to the development of anxiety and depression (Turan & Karaaslan, 2020). Nevertheless, a case–control study of children aged 8–16 years found that the prevalence of depression was higher in the adequate iodine intake group than in the excessive group. Further, Wang et al. found that iodine supplementation during pregnancy had no significant effect on postpartum depression, though the difference in the Edinburgh postnatal depression scale (Wang et al., 2020). These differences in results may be attributed to the fact that the effects of relevant confounding factors, such as protein intake, have not been fully considered.

Several epidemiological studies have explored the relationship between dietary protein and depression, discovering higher protein intake was associated with a reduced risk of depressed mood (Ciarambino et al., 2011; Li et al., 2020; Rubio‐López et al., 2016; Wolfe et al., 2011). However, previous studies have only evaluated these as separate factors. And there are no relevant clinical studies that have been performed investigating the effect of protein intake on the relationship between iodine levels and the risk of depression symptoms. It is well known that almost all iodine ingested by the body is excreted in the urine (Vejbjerg et al., 2009), so most of the iodine is present in the body in the form of urinary iodine. Several studies have shown that urinary iodine can be used as a surrogate measure of dietary iodine content (Andersen et al., 2015; Mikulska et al., 2021). Similarly, the World Health Organization (WHO) recommends the use of urine assessments to measure people's iodine levels (Peniamina et al., 2019). Therefore, the purpose of this study was to investigate the association between UIC (urinary iodine concentrations) and CRD (clinically relevant depression) in different protein levels of US adults. It was hypothesized that the coexistence of inadequate UIC and adequate protein intake might have an antagonistic effect on lowering clinically relevant depression, thereby reducing the prevalence of depressive symptoms.

## 2 MATERIALS AND METHODS

### 2.1 Study population

**FIGURE 1:**
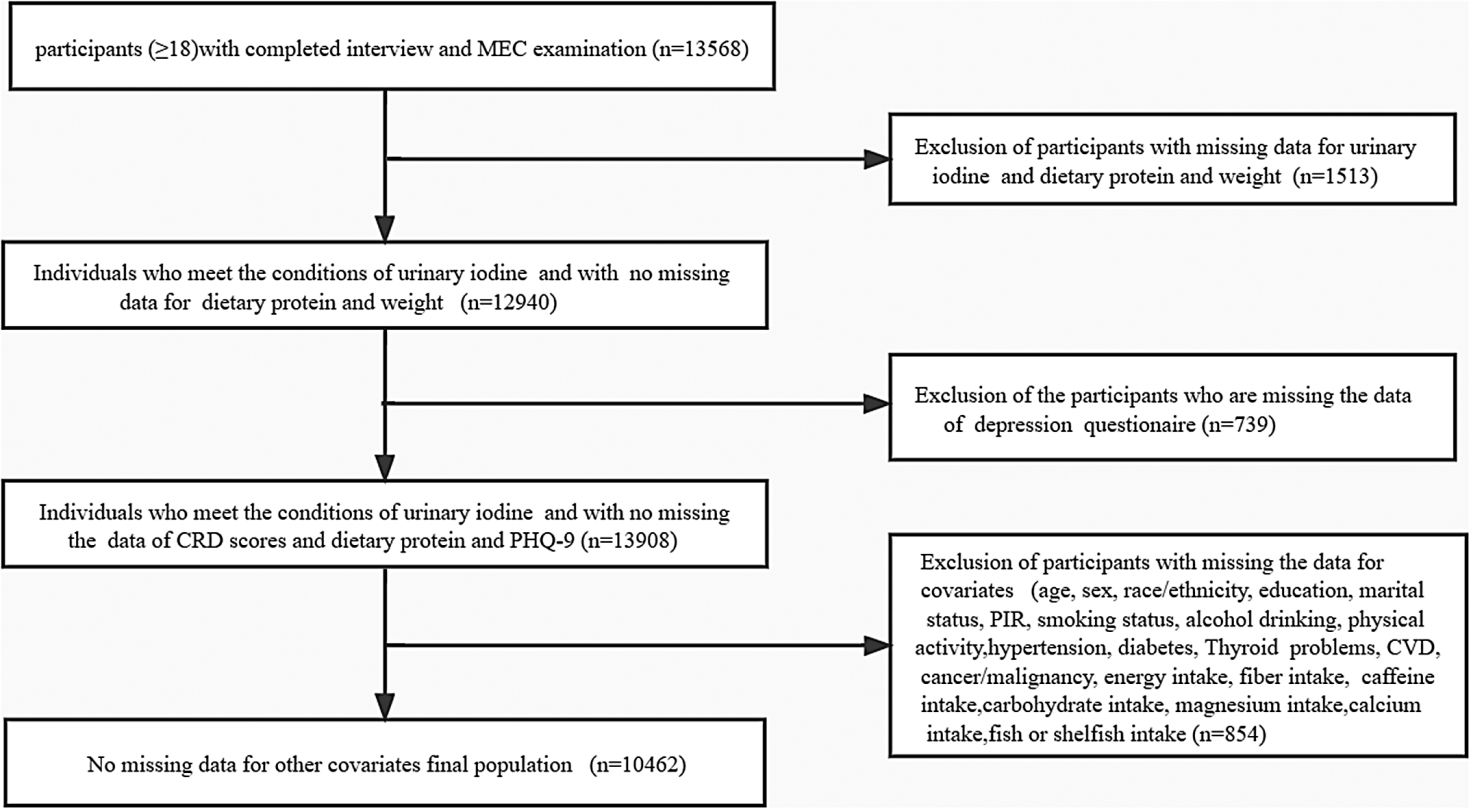
The flowchart of inclusion and exclusion in the study.

The NHANES database, which is based on a stratified, complex, multistage, and probability cluster designed to obtain data from nationally representative samples in the US, is mobilized by the National Center of Health Statistics of the Centers for Disease Control and Prevention (Huang et al., 2021; Inoue et al., 2018). A comprehensive household interview was conducted to obtain information on demographics and health history. Using a mobile examination center (MEC) to perform a physical examination and collect blood samples. It consists of demographic data, dietary interviews (24‐h dietary recall), questionnaire information, laboratory tests, and examinations administered by trained staff (Centers for Disease Control and Prevention, 2022b). The NHANES data for 2007–2018 contain all iodine and CRD. In these populations, we removed the data (1513) for missing depression PHQ‐9 scores, protein intake, and UIC. Further, we excluded those who were not eligible for data analysis because of missing information on key demographics such as age, gender, race/ethnicity, smoking status, and physical activity, leaving 10,462 participants as the final analytic sample. Additional details of the study sampling and exclusion criteria are shown in Figure 1. This study was supported by the National Center for Health Statistics Research Ethics Review Board, and the ethics approval numbers are Protocol #2005–06, Protocol #2011–17, and Protocol #2018–01. You can find it at this website: NCHS Ethics Review Board Approval (cdc.gov). Everyone provided informed consent. And all the data used in the manuscript can be available on the website: https://wwwn.cdc.gov/nchs/nhanes/search/default.aspx.

### 2.2 Outcome ascertainment

Depressive symptoms were evaluated using a nine‐item self‐report rating scale, the Patient Health Questionnaire (PHQ‐9) (Spitzer et al., 1999). The PHQ‐9 is consistent with the symptom management model, in which symptoms are regarded as subjective experiences involving changes in biopsychosocial function and cognition (Peppard et al., 2019). The reliability coefficient Cronbach's alpha of the PHQ‐9 was 0.84, with a sensitivity of 88% and specificity of 88% (Kroenke et al., 2001). During the home interview, participants were asked questions such as the following: “Over the last 2 weeks, how often have you been bothered by the following problems: little interest or pleasure in doing things?” or “Do you feel bad about yourself ‐or that you are a failure or have let yourself or your family down?” Respondents who answered “yes” to this question were considered as having received mental health care, and respondents who answered “no” were considered as having received no mental health care. Participants who refused to answer or who answered “do not know” were excluded. Each event was assigned 0–3 points, and the overall score ranged from 0 to 27. Participants were divided into two groups based on their overall scores: those with scores greater than 10 were classified as depressed and those with scores less than or equal to 10 were classified as nondepressed (Medici et al., 2015; Spitzer et al., 1999).

### 2.3 Urinary iodine concentrations

In NHANES, urinary iodine concentrations were determined by inductively coupled plasma dynamic reaction cell mass spectrometry (ICP‐DRC‐MS), and measurements of urinary iodine were taken in one‐third of the subsamples of people aged 6 years and older (Centers for Disease Control and Prevention, 2022c). According to the WHO guidelines, urine iodine (μg/L) was divided into four groups from low to high: low UIC, <100 μg/L; normal UIC, 100–299 μg/L; high UIC, 300–399 μg/L; and very high UIC, ≥400 μg/L (Inoue et al., 2018).

### 2.4 Dietary protein intake

Trained interviewers assessed dietary intake through two 24‐h dietary recall interviews. The first dietary recall interview was collected in person at the MEC, and the second interview was collected by telephone 3–10 days later. Participants were provided with measurement guides and food model booklets to help them report the amount of food consumed during the interview. Dietary studies from the United States Department of Agriculture (USDA) Food and Nutrient Database were used to calculate nutrient‐related intakes. Dietary protein on the first day was used in this study, and protein intake (g/kg/d) was obtained by dividing protein intake (g/d) by body weight (kg). Daily protein intake was divided into high intake (≥0.8 g/kg/d) and low intake (<0.8 g/kg/d) based on 0.8 g/kg/d. In large‐scale surveys, the 24‐h recall method is mostly used to determine dietary intake. This method of data collection by NHANES is supported by the consensus of a panel of experts in a regular evaluation workshop (Ahluwalia et al., 2016).

### 2.5 Covariate assessments

The selection of covariates was based on previous literature, clinical experience, and the statistical significance of reason. Standardized questionnaires were used to collect information on age, sex, race/ethnicity, education, marital status, annual family income, smoking status, alcohol intake, physical activity, energy intake, caffeine intake, fish or shellfish intake, fiber intake, magnesium intake, calcium intake, protein intake, and carbohydrate intake. Race/ethnicity was categorized as Mexican American, non‐Hispanic white, non‐Hispanic black, or other. Education was classified as below high school, high school grade/general education development diploma or equivalent (GED), or above high school (Francis et al., 2021). Marital status was categorized as married/living with a partner, widowed, divorced/separated, or never married. Two categories of the annual family income were considered: “income < $20000”, and “income ≥ $20000”. Smoking status was defined as the numbers and timeline of cigarettes in life (never, smoked less than 100 cigarettes; former, smoked more than 100 cigarettes in life and smoke not at all now; now, smoked more than 100 cigarettes in life and smoke some days or every day) (Ruan et al., 2022). Participants were categorized as mild, moderate, and heavy based on the number of drinks per day he/she had drunk. Participants who are “mild” were considered to be drinking alcohol ≤1 drink in women and ≤2 drinks in men; Participants who are “moderate” were considered to be drinking alcohol ≤2 drinks in women and ≤3 drinks in men. And individuals who had drunk ≥3 drinks in women and ≥4 drinks in men were thought “heavy” (Rattan et al., 2022). The data on energy intake, caffeine intake, fish or shellfish intake, fiber intake, magnesium intake, calcium intake, protein intake, and carbohydrate intake were also collected through the dietary recall interview (Centers for Disease Control and Prevention, 2022a), respectively. Physical activity was calculated based on the amount of exercise per day multiplied by the Met (metabolic equivalent task) of exercise intensity (Lee et al., 2016), where the amount of exercise per month and per week is obtained by dividing by the corresponding number of days. Diabetes was defined as a diagnosis from a doctor or other health professional, 2‐h OGTT blood glucose (mmol/l) ≥ 11.1, random blood glucose (mmol/L) ≥ 11.1, fasting glucose (mmol/l) ≥ 7.0, HbA1c (%) >6.5, or use of medication or insulin. Hypertension, cancer/malignancy, and serious cardiovascular disease (coronary heart disease/heart attack/stroke) were assessed by asking participants about their diagnoses (Holingue et al., 2018; Reinstatler et al., 2017; Zhang et al., 2022). Thyroid problems were defined as those people who have been diagnosed with thyroid disease or population who use thyroxine tablets. In this study, the participants who disposed to “refused” and “do not know” were treated as missing.

### 2.6 Statistical analysis

The study was designed in strict accordance with the STROBE guidelines (Vandenbroucke et al., 2007). All analyses were performed using the statistical software packages R (http://www.r‐project.org; version 4.2.2, The R Foundation) and Free Statistics software version 1.7 (Yang et al., 2021). We used sample weights provided by the 2007–2018 NHANES, and the present data can represent a sample population of 74,676,948. Continuous normal variables were expressed as weighted mean ± standard deviation and t‐tests were used to compare differences between groups. Categorical variables were expressed as frequencies and percentages and compared using Rao–Scott's χ^2^ test. A two‐sided *p*‐value less than 0.05 indicates a denoted statistically significant difference. Logistic regression model was used to calculate the odds ratio (OR) and 95% confidence interval (CI) for the relationship between CRD prevalence and UIC, and the categorical normal group of UIC (100–300 μg/L) was used as a reference. Subgroup analysis of protein intake was performed using a stratified weighted multivariate logistic regression model. For these models, we used untuned and adjusted models. First and foremost, we adjusted for age and sex in Model 1. We further adjusted for hypertension, diabetes, thyroid problems, CVD, and the covariates of Model 1 besides (Model 2). We also adjusted for education level, marital status, race/ethnicity, smoking status, alcohol consumption, and physical activity and Model 2 (Model 3). Finally, the dietary data including energy intake, caffeine intake, fish or shellfish intake, fiber intake, magnesium intake, calcium intake, and carbohydrate intake were further adjusted in Model 4. We investigated the continuous association between UIC and depression by fitting a restricted cubic spline model with four segments at the 5th, 35th, 65th, and 95th percentiles of UIC (log10) (Greenland, 1995). In addition, we examined the multiplicative interaction of protein intake and UIC on the prevalence of CRD and calculated the interaction *p*‐value using the Wald test.

### 2.7 Participants and public involvement

Participants were not involved in our research design, reporting, and dissemination plans of our research.

## 3 RESULTS

### 3.1 Participant characteristics according to protein intake

In this study, we selected six continuous NHANES cycles (2007–2008, 2009–2010, 2011–2012, 2013–2014, 2015–2016, 2017–2018) and focused on 13,568 adults with completed interviews and MEC examination in the US (≥18 years). Among the 10,462 participants in the study, 5285 males and 5177 females were recruited, and a total of 947 participants (9.1%) had CRD, and 9515 (90.1%) did not have CRD. Based on the weighted analyses, the mean age of the 10,462 participants was 47.0 years (range, 46.7–47.3 years) and those with education of above high school accounted for 51%, and most of the participants were non‐Hispanic white (45.5%). Participants with lower protein intake were more likely to be female, non‐Hispanic white, with lower annual household income, nonhypertensive, nondiabetic, CRD, and with lower met of physical activity. For dietary factors, the intake of energy, fiber, carbohydrate, calcium, magnesium, and caffeine was higher in participants whose intake of protein was high. Smoking habits, annual household income, cancer, or malignancy did not differ by protein intake. The baseline characteristics of the participants are summarized in Table 1.

**TABLE 1:** Baseline characteristics of participants according to protein intake groups.

Characteristics	Protein intake(g/kg/d)			
	Total	<0.8 (4200)	≥ 0.8 (6200)	*p‐*Value
Gender				<.001
Female	5177 (49.5)	2455 (61.8)	2722 (44.9)	
Male	5285 (50.5)	1745 (38.2)	3540 (55.1)	
Ethnicity				<.001
Non‐Hispanic black	2125 (20.3)	1066 (13.6)	1059 (8.5)	
Non‐Hispanic white	4758 (45.5)	1877 (68.5)	2881 (70.0)	
Mexican American	1662 (15.9)	585 (7.5)	1077 (9.1)	
Other	1917 (18.3)	672 (10.4)	1245 (12.4)	
Marital				<.001
Divorced/separated	1498 (14.3)	672 (14.6)	826 (11.9)	
Never married	1873 (17.9)	716 (19.9)	1157 (19.3)	
Married/Living with a partner	6273 (60.0)	2393 (58.3)	3880 (64.2)	
Widowed	818 (7.8)	419 (7.2)	399 (4.6)	
Annual family income ($)				.921
<20,000	10,130 (96.8)	4067 (97.8)	6063 (97.8)	
≥20,000	332 (3.2)	133 (2.2)	199 (2.2)	
Education				.004
Below high school	2681 (25.6)	1156 (18.0)	1525 (15.7)	
High School Grade/GED or Equivalent	2441 (23.3)	1016 (24.3)	1425 (22.6)	
Above high school	5340 (51.0)	2028 (57.7)	3312 (61.7)	
Smoke				.541
Former	2652 (25.3)	1101 (25.3)	1551 (24.6)	
Never	5595 (53.5)	2225 (52.6)	3370 (54.2)	
Now	2215 (21.2)	874 (22.1)	1341 (21.2)	
Alcohol				<.001
Mild	3743 (35.8)	1342 (36.9)	2401 (41.3)	
Moderate	3337 (31.9)	1280 (31.9)	2057 (35.8)	
Heavy	3382 (32.3)	1578 (31.2)	1804 (22.9)	
Physical activity	545.82 ± 13.50	464.96 ± 18.44	594.68 ± 15.88	<.001
Hypertension				<.001
No	5960 (57)	2025 (55.0)	3935 (67.2)	
Yes	4502 (43)	2175 (45.0)	2327 (32.8)	
DM				<.001
No	8454 (80.8)	3148 (80.8)	5306 (88.7)	
Yes	2008 (19.2)	1052 (19.2)	956 (11.3)	
CVD				<.001
No	9355 (89.4)	3588 (88.3)	5767 (94.1)	
Yes	1107 (10.6)	612 (11.7)	495 (5.9)	
Cancer				.088
No	9421 (90.0)	3729 (88.9)	5692 (90.7)	
Yes	1041 (10.0)	471 (11.1)	570 (9.3)	
Thyroid disease				<.001
No	9362 (89.5)	3640 (85.3)	5722 (90.6)	
Yes	1100 (10.5)	560 (14.7)	540 (9.4)	
CRD				<.001
No	9515 (90.9)	3699 (88.9)	5816 (93.9)	
Yes	947 (9.1)	501 (11.1)	446 (6.1)	
UIC (μg/L)				.007
100–299	4989 (47.7)	2059 (48.6)	2930 (44.5)	
<100	3557 (34.0)	1355 (33.5)	2202 (37.7)	
300–399	772 (7.4)	296 (7.8)	476 (7.3)	
≥400	1144 (10.9)	490 (10.1)	654 (10.5)	
Fish/Shellfish				<.001
No	2062 (19.7)	960 (21.9)	1102 (16.7)	
Yes	8400 (80.3)	3240 (78.1)	5160 (83.3)	
Age	46.99 ± 0.33	48.81 ± 0.40	45.89 ± 0.40	<.001
BMI (kg.m^2^)	28.89 ± 0.10	31.73 ± 0.15	27.18 ± 0.11	<.001
Energy intake	2144.63 ± 13.82	1537.44 ± 12.75	2511.53 ± 17.36	<.001
Carbohydrate intake	255.43 ± 1.58	199.24 ± 1.87	289.38 ± 2.30	<.001
Fiber intake	16.73 ± 0.27	12.43 ± 0.22	19.32 ± 0.30	<.001
Calcium intake	967.43 ± 11.53	673.49 ± 8.02	1145.04 ± 12.26	<.001
Magnesium intake	302.21 ± 3.61	215.05 ± 2.48	354.87 ± 3.66	<.001
Caffeine intake	180.31 ± 5.02	168.09 ± 6.58	187.70 ± 5.64	.005

*Note*: Continuous normal variables are expressed as weighted mean ± standard deviation, *T*‐tests were used to compare differences between two groups. Categorical variables are expressed as frequencies and percentages and compared using Rao–Scott's χ^2^ test.

Abbreviations: BMI, body mass index; CRD, clinically relevant depression; CVD, severe cardiovascular diseases; GED, general education development diploma; MET, metabolic equivalents of task; UIC, urinary iodine concentration.

### 3.2 Distribution of UIC in the CRD group by protein intake

**FIGURE 2:**
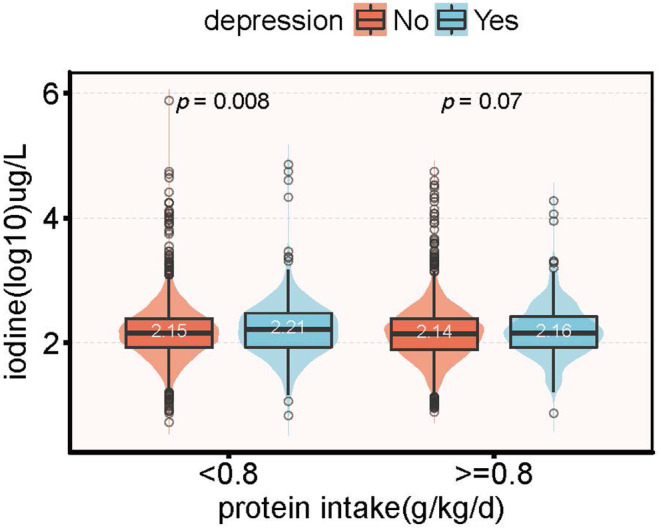
Distribution of urinary iodine in patients with depressive symptoms grouped by protein intake.

Figure 2 shows the difference in UIC levels between CRD participants and non‐CRD participants. In the high protein, there was no statistical difference in UIC levels between the CRD and non‐CRD populations (2.16 vs. 2.14, *p* = .07). However, in the low‐protein group, the UIC levels were significantly higher in the depressed population than in the nondepressed population (2.21 vs. 2.15, *p* = .008).

### 3.3 Multivariate logistic regression analysis of urinary iodine and depression

The OR and corresponding 95% CI of the risk for CRD according to UIC (log10) and four UIC groups are summarized in Table 2. When UIC was a continuous value, a multivariate regression model was used to adjust for other possible confounders, including demographic factors, chronic illness, lifestyle habits, and dietary factors, and it was found that with each 1og‐unit increase in UIC level, there was a corresponding 36% increase in the probability of developing depression. The same trend was observed in the category very high UIC group (UIC ≥400 μg/L) after adjustment for all relevant covariates: participants who had very high UIC had a 42% increased risk of the probability of developing CRD compared with the normal group (OR: 1.42, [95% CI: 1.038–1.933]) and the effect estimates also above 1 in the other three different adjustment models in Table 2.

**TABLE 2:** Association between UIC and CRD among participants in the NHANES 2007–2016.

Variable	Crude model		Model 1		Model 2		Model 3		Model 4	
	OR 95% CI	*p*‐Value	OR 95% CI	*p*‐Value	OR 95% CI	*p*‐Value	OR 95% CI	*p*‐Value	OR 95% CI	*p*‐Value
UIC (log10)	1.320 (0.974, 1.775)	.073	1.423 (1.053, 1.922)	.022	1.301 (0.978, 1.730)	.071	1.375 (1.035, 1.827)	.029	1.360 (1.026, 1.795)	.033
UIC group (μg/L)										
100–299	Reference		Reference		Reference		Reference		Reference	
<100	1.016 (0.793, 1.301)	.900	0.963 (0.754, 1.228)	.756	1.000 (0.782, 1.279)	1.000	0.979 (0.759, 1.264)	.870	1.003 (0.777, 1.295)	.981
300–399	1.302 (0.879, 1.929)	.185	1.326 (0.901, 1.950)	.150	1.286 (0.885, 1.867)	.183	1.342 (0.918, 1.962)	.126	1.355 (0.922, 1.992)	.120
≥400	1.362 (1.011, 1.835)	.042	1.390 (1.030, 1.875)	.032	1.358 (1.008, 1.829)	.044	1.416 (1.038, 1.933)	.029	1.422 (1.039, 1.948)	.029

*Note*: Calculation using multivariate logistic regression analysis was performed.

Model 1: Adjusted for age and sex;

Model 2: Adjusted for age, sex, hypertension, diabetes, thyroid problems, and CVD;

Model 3: Adjusted for age, sex, hypertension, diabetes, thyroid problems, CVD, education level, marital status, race/ethnicity, smoking status, alcohol consumption, and physical activity;

Model 4: Adjusted for age, sex, hypertension, diabetes, thyroid problems, CVD, education level, marital status, race/ethnicity, smoking status, alcohol consumption, physical activity, energy intake, caffeine intake, fish or shellfish intake, fiber intake, magnesium intake, calcium intake, and carbohydrate intake.

### 3.4 Protein intake affects the relationship between UIC and the risk of CRD

Urinary iodine concentration levels and CRD showed different performances with protein intake. In individuals with low protein intake (<0.8 g/kg/d), no correlation between continuous UIC and CRD was observed inside all models, both before and after model adjustment. However, an increased risk of depression was observed in the UIC subgroup, whether in the low UIC group, the high UIC group, or the very high UIC group compared with the normal UIC group in Table 3 and the trend (*p* < .001).

**TABLE 3:** Logistic regression between UIC and CRD among participants in the NHANES 2007–2016 according to protein intake.

Variable	Crude model		Model 1		Model 2		Model 3		Model 4	
	OR 95% CI	*p*‐Value	OR 95% CI	*p*‐Value	OR 95% CI	*p*‐Value	OR 95% CI	*p*‐Value	OR 95% CI	*p*‐Value
*Low‐protein intake group (<0.8 g/kg/d)*										
UIC (μg/L)										
100–299	Ref		Ref		Ref		Ref		Ref	
<100	1.497 (1.075, 2.083)	.018	1.426 (1.033, 1.970)	.032	1.460 (1.046, 2.037)	.027	1.476 (1.036, 2.104)	.032	1.470 (1.017, 2.124)	.041
300–399	2.153 (1.176, 3.941)	.014	2.209 (1.202, 4.060)	.011	2.168 (1.195, 3.936)	.012	2.131 (1.158, 3.922)	.016	2.283 (1.268, 4.110)	.007
≥400	1.575 (1.139, 2.176)	.007	1.669 (1.221, 2.282)	.002	1.643 (1.203, 2.244)	.002	1.587 (1.139, 2.211)	.007	1.631 (1.175, 2.263)	.004
*p* for trend		<.001		<.001		<.001		.002		<.001
*High‐protein intake group (≥0.8 g/kg/d)*										
UIC (μg/L)										
100–299	Ref		Ref		Ref		Ref		Ref	
<100	0.732 (0.525, 1.019)	.065	0.700 (0.498, 0.983)	.04	0.723 (0.517, 1.011)	.058	0.716 (0.522, 0.981)	.038	0.728 (0.533, 0.995)	.046
300–399	0.702 (0.439, 1.123)	.137	0.712 (0.445, 1.139)	.154	0.703 (0.429, 1.151)	.158	0.770 (0.461, 1.289)	.314	0.755 (0.446, 1.277)	.288
≥400	1.235 (0.775, 1.969)	.370	1.236 (0.775, 1.971)	.369	1.206 (0.755, 1.926)	.428	1.251 (0.771, 2.031)	.358	1.240 (0.759, 2.026)	.384
*p* for trend		.881		.893		.943		.79		.831
*p* for interaction		.131		.122		.099		.128		.148

*Note*: Model 1: Adjusted for age and sex.

Model 2: Adjusted for age, sex, hypertension, diabetes, thyroid problems, and CVD.

Model 3: Adjusted for age, sex, hypertension, diabetes, thyroid problems, CVD, education level, marital status, race/ethnicity, smoking status, alcohol consumption, and physical activity.

Model 4: Adjusted for age, sex, hypertension, diabetes, thyroid problems, CVD, education level, marital status, race/ethnicity, smoking status, alcohol consumption, physical activity, energy intake, caffeine intake, fish or shellfish intake, fiber intake, magnesium intake, calcium intake, and carbohydrate intake.

**FIGURE 3:**
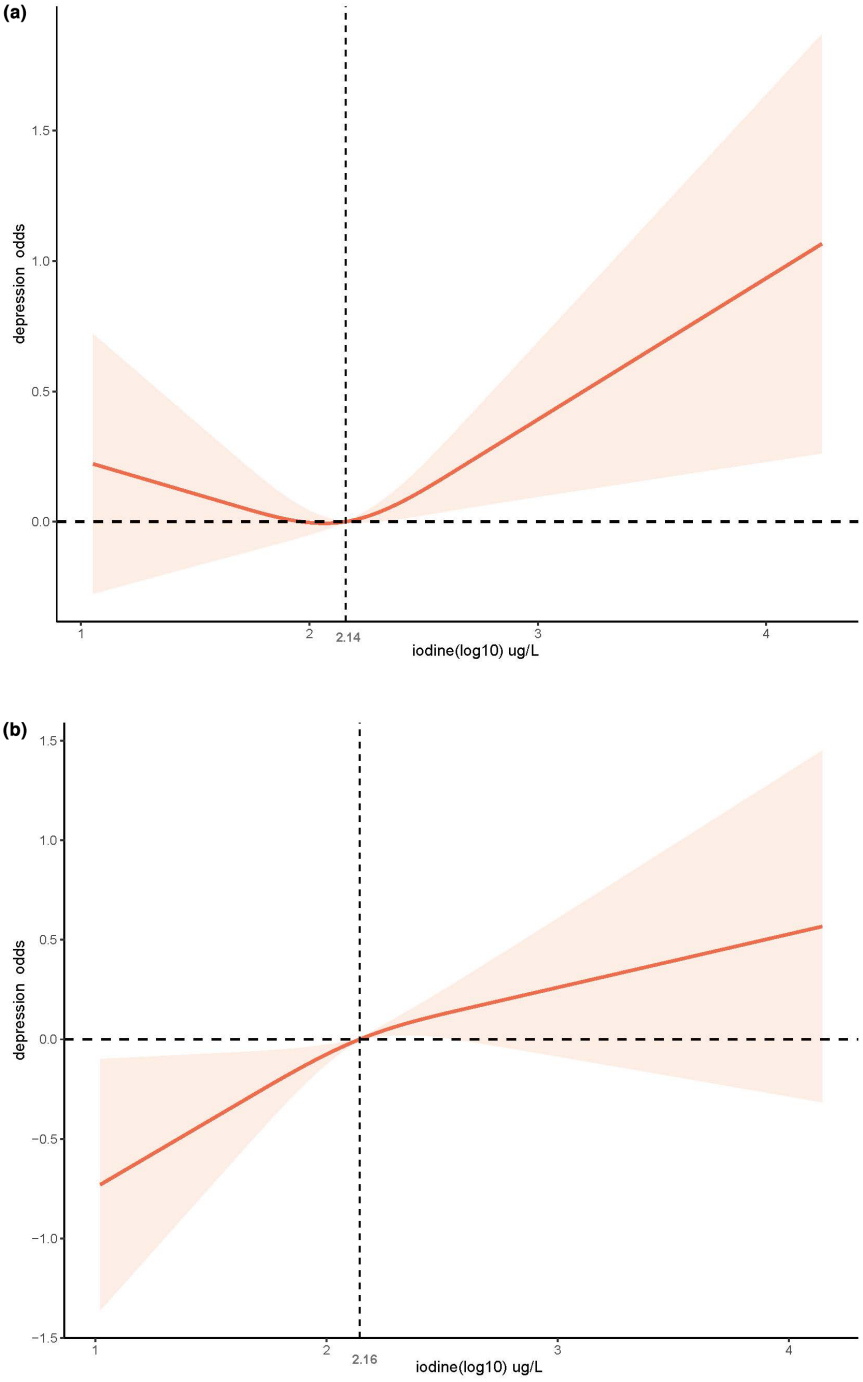
Restricted cubic spline plot of the association between UIC and CRD (a) in low‐protein population (b) in high‐protein population adjusted for age, sex, race/ethnicity, education, marital status, the annual family income, smoking status, alcohol intake, physical activity, hypertension, diabetes, thyroid problems, CVD, energy intake, caffeine intake, fish or shellfish intake, fiber intake, magnesium intake, calcium intake, protein intake, and carbohydrate intake.

For individuals with high protein intake (≥0.8 g/kg/d), continuous UIC showed an inverse association with CRD. When UIC was categorized, the association between UIC and CRD was also not statistically significant in unadjusted analyses. However, after adjusting for all confounders, compared with normal UIC, the low UIC group (<100 μg/L) showed would reduce the incidence of CRD (in models 1, 3, and 4). At the same time, no significant association was observed between UIC and CRD at high UIC and very high group, though the value of OR was over 1 and statistical results were robust among all the models. In addition, a restricted cubic spline showed a near U‐shaped (nonlinear *p* = .042) relationship between UIC and CRD in low‐protein population and a linear (nonlinear *p* = .392) relationship of it in high‐protein people in Figure 3.

## 4 DISCUSSION

The present study evaluated a representative sample of American adults, and we found a positive association between UIC and the risk of CRD, even though adjusted for potentially important confounders. We also found that in the fully adjusted model, the ORs for CRD in participants with low UIC compared to the normal UIC group were 0.73 (95% CI: 0.533, 0.995) in the high protein intake group. However, it may be a risk factor for CRD whether UIC is low, high, or very high group compared with the normal UIC group in the low protein (≤0.8 g/kg/d) population and a nonlinear negative relationship in the group. Therefore, a strategy that includes low UIC and high fiber intake may be more effective in reducing the incidence of CRD.

To the best of our knowledge, studies have investigated the association between iodine and depression. There are three studies that supported our study. A follow‐up study of pregnant women in the Liaoning Province of China showed that pregnant women taking iodine‐containing vitamin supplementation had higher depression scores than those not taking iodine‐containing vitamins and those receiving no vitamins (Wang et al., 2020). Another large cohort study from Norwegian involving 2792 pregnant women also found an increased risk of emotional distress during pregnancy with iodine supplement use compared to the control group (Brantsæter et al., 2022). Furthermore, a recent study conducted by Chen et al. found that high urinary iodine group was associated with a higher prevalence of depressive symptoms than the normal urine iodine group in the US female of 40–59 population (Chen et al., 2022).

However, a case–control study of Chinese adolescents aged 8–16 years from the Pediatric Health Care Outpatient Center of Zao Zhuang Children's Hospital showed that adolescents with depressive symptoms had significantly lower urinary iodine levels than control patients (Huang et al., 2019). Moreover, a study of the relationship between iodine levels and anxiety and depression in patients with nodular goiter (ENG) with normal thyroid function found that the Beck Anxiety Inventory (BAI) scores were significantly higher in the low UIC group than in the high UIC group (*p* = .007) (Turan & Karaaslan, 2020). This inconsistency may be due to confounding factors such as protein intake, differences in the study population, and sample size.

Amino acids in proteins have been reported to affect mood and cognitive function (Wurtman & Wurtman, 1995). Tryptophan can be converted into serotonin (5‐HT) and thus has similar effects to antidepressants (Wong & Ong, 2001). Tyrosine, on the other hand, has an effect on mood by converting the neurotransmitter into dopamine (Rao et al., 2008). In addition, other amino acid compounds, such as peptides, are thought to affect brain metabolism (Nagasawa et al., 2012). And an experimental animal study found that myostatin can reduce the stress induced by forced swimming tests (Tomonaga et al., 2008). A study from Japan suggests that low protein intake may be associated with a higher prevalence of depressive symptoms among Japanese male workers (Nanri et al., 2014). Another cross‐sectional study from Finland on patients with type 1 diabetes reported that it was protein rather than carbohydrates and fat was associated with lower levels of depressive symptoms (Ahola et al., 2018). However, a 10‐year follow‐up of a national cohort study found a positive association between protein intake and major depressive mood in US women (Wolfe et al., 2011). In our study, high protein intake was found to reduce the risk of depression in the low UIC population, but a higher risk of CRD with UIC was observed in the low‐protein group.

There are some biological reasons to support our result. Iodine is the main component of the thyroid hormones thyroxine T3 and triiodothyronine T4 (Grieco et al., 2020). The 5‐hydroxytryptophan hypothesis is the more recognized hypothesis in the biochemical mechanisms of depression, and the systemic disorder of depression can be triggered by different factors of the vulnerable 5‐hydroxytryptophan system (Dell'Osso et al., 2016; Israelyan et al., 2019; Yue et al., 2019). Iodine affects thyroid function, and thyroxine can predispose the neurotransmission of norepinephrine adenosine in the body, which can affect β‐adrenergic receptors or 5‐hydroxy chromogranin in the body through this pathway, thus affecting the onset of depression. It is important to note that both protein and iodine are inseparable from the proper functioning of the thyroid gland. Thyroid hormones are formed by adding iodine to the amino acid tyrosine, which usually requires transport in the bloodstream via carrier proteins. And in the case of low protein, it is not conducive to normal thyroid function (Ingenbleek & Beckers, 1978). In addition, it has been shown that low protein can exacerbate thyroid damage from excess iodine alone (Gao et al., 2013). Therefore, it is possible for a high protein to help to alleviate the harm caused by iodine abnormalities to the thyroid gland, which affects the occurrence of depression.

This study has several strengths. First, the large sample was recruited from a clinically relevant population. Second, this is the first study to examine the association between UIC and CRD according to protein intake in the NHANES database of the US population. Third, we adjusted the model for a variety of potential confounders, including demographic, lifestyle, chronic disease, and dietary factors, and our primary results persisted after adjustment for these factors. In addition, we performed a dose–response analysis to assess the relationship between UIC and CRD in different protein states. There are also some limitations to this study. First and foremost, this study was a cross‐sectional observational study; therefore, the association may not lead to direct causation. Meanwhile, the relationship we studied may have been influenced by other confounding factors, which we have not adjusted. Finally, there were biases in reporting and recall. Because our dietary intake came from a 24‐h recall questionnaire. However, this method is feasible, and to ensure the feasibility of NHANES as a data collection method, periodic assessments of data collection procedures and expert workshops are held for consensus (Ahluwalia et al., 2016).

## 5 CONCLUSIONS

In general, we found that whether a person is high in protein may have a great impact on the association between UIC and CRD risk. Combining lower UIC and high protein intake may help to reduce the prevalence of CRD, which would have significant implications for managing patients with CRD in the clinical setting.

## AUTHOR CONTRIBUTIONS

**Xue Kong:** Conceptualization (equal); data curation (equal); formal analysis (equal); resources (equal); writing – original draft (equal); writing – review and editing (equal). **Xia Shen:** Data curation (equal); formal analysis (equal); methodology (equal); resources (equal); software (equal); writing – original draft (equal); writing – review and editing (equal). **Long Yang:** Formal analysis (equal); validation (equal); writing – review and editing (equal). **Yuan‐Yuan Liu:** Investigation (equal); writing – review and editing (equal). **Xue Gu:** Investigation (equal); writing – review and editing (equal). **Yan Kong:** Supervision (equal).

## CONFLICT OF INTEREST STATEMENT

The authors declare that they have no conflict of interest.

## INSTITUTIONAL REVIEW BOARD STATEMENT

This study was supported by the National Center for Health Statistics Research Ethics Review Board, and the ethics approval numbers are Protocol #2005–06, Protocol #2011–17, and Protocol #2018–01. You can find it at this website: NCHS Ethics Review Board Approval (cdc.gov).

## INFORMED CONSENT STATEMENT

This study is an analysis of the publicly available NHANES data. Informed consent was obtained from NHANES participants by the National Center for Health Statistics Research Ethics Review Board.

## Data Availability

All the data are available in the public and they were used in the manuscript. The data can be availed from the website: https://wwwn.cdc.gov/nchs/nhanes/search/default.aspx
